# (−)-Englerin A-evoked Cytotoxicity Is Mediated by Na^+^ Influx and Counteracted by Na^+^/K^+^-ATPase*

**DOI:** 10.1074/jbc.M116.755678

**Published:** 2016-11-14

**Authors:** Melanie J. Ludlow, Hannah J. Gaunt, Hussein N. Rubaiy, Katie E. Musialowski, Nicola M. Blythe, Naveen S. Vasudev, Katsuhiko Muraki, David J. Beech

**Affiliations:** From the ‡ Leeds Institute of Cardiovascular and Metabolic Medicine, School of Medicine, University of Leeds, Leeds LS2 9JT, United Kingdom and; the § School of Pharmacy, Aichi-Gakuin University, 1-100 Kusumoto, Chikusa, Nagoya 464-8650, Japan

**Keywords:** calcium, cancer, cell death, Na^+^/K^+^-ATPase, transient receptor potential channels (TRP channels), ouabain, sodium

## Abstract

(−)-Englerin A ((−)-EA) has a rapid and potent cytotoxic effect on several types of cancer cell that is mediated by plasma membrane ion channels containing transient receptor potential canonical 4 (TRPC4) protein. Because these channels are Ca^2+^-permeable, it was initially thought that the cytotoxicity arose as a consequence of Ca^2+^ overload. Here we show that this is not the case and that the effect of (−)-EA is mediated by a heteromer of TRPC4 and TRPC1 proteins. Both TRPC4 and TRPC1 were required for (−)-EA cytotoxicity; however, although TRPC4 was necessary for the (−)-EA-evoked Ca^2+^ elevation, TRPC1 was not. TRPC1 either had no role or was a negative regulator of Ca^2+^ entry. By contrast, both TRPC4 and TRPC1 were necessary for monovalent cation entry evoked by (−)-EA, and (−)-EA-evoked cell death was dependent upon entry of the monovalent cation Na^+^. We therefore hypothesized that Na^+^/K^+^-ATPase might act protectively by counteracting the Na^+^ load resulting from sustained Na^+^ entry. Indeed, inhibition of Na^+^/K^+^-ATPase by ouabain potently and strongly increased (−)-EA-evoked cytotoxicity. The data suggest that (−)-EA achieves cancer cell cytotoxicity by inducing sustained Na^+^ entry through heteromeric TRPC1/TRPC4 channels and that the cytotoxic effect of (−)-EA can be potentiated by Na^+^/K^+^-ATPase inhibition.

## Introduction

(−)-Englerin A ((−)-EA)^2^ is a sesquiterpene from the bark of the African plant *Phyllanthus engleri*. Its isolation was first reported in 2009 alongside an NCI60 human tumor cell line screen demonstrating its rapid cytotoxic potential with potency in particular against renal cell carcinoma cell lines (1). Currently approved treatments for renal cell carcinoma are of limited efficacy and are often toxic. Therefore, there has been keen interest in the discovery of (−)-EA and methods for its synthesis (2–5). A larger screen revealed that (−)-EA kills cells from a wide range of lineages but again supported the absence of adverse effects on normal, non-cancerous cells (6). Efforts to identify the cellular target of (−)-EA have suggested that it activates PKCθ and the Ca^2+^-permeable non-selective channels formed by assembly of transient receptor potential canonical (TRPC) proteins TRPC4 and TRPC5 (6–8). PKCθ was involved in (−)-EA-induced cell death in the 786-0 renal carcinoma cell line (8) but was barely detectable in other (−)-EA-sensitive cells (A498 renal carcinoma and A637 Ewing's sarcoma-derived cell lines) (6, 7), and its proposed mechanism of action, promoting dependence on glucose while simultaneously starving cells of glucose (8), involves relatively slow gene regulatory events. Activation of an ion channel would be more consistent with the rapid (<1 h) onset of cell death induced by (−)-EA (7), and analysis of >500 well characterized cancer cell lines revealed that TRPC4 mRNA abundance is the feature best correlated with sensitivity to (−)-EA (6). In support, knockdown of TRPC4 in A498 and A673 cells offered protection against (−)-EA-induced cell death (6). The related TRPC5 protein is, in contrast, rarely overexpressed in tumor cell lines (6). Notably, the latter study identified a few exceptions, cells with enhanced TRPC4 but low sensitivity to (−)-EA, indicating that up-regulation of TRPC4 is not the sole factor imparting (−)-EA sensitivity. Although ion channels are increasingly suggested to have important roles in cancer development and progression, inhibitors, rather than activators, represent the more obvious approach for drug discovery. Therefore, it is important to better understand how activation of TRPC4-containing channels by (−)-EA results in rapid cancer cell death.

## Results

**FIGURE 1.**
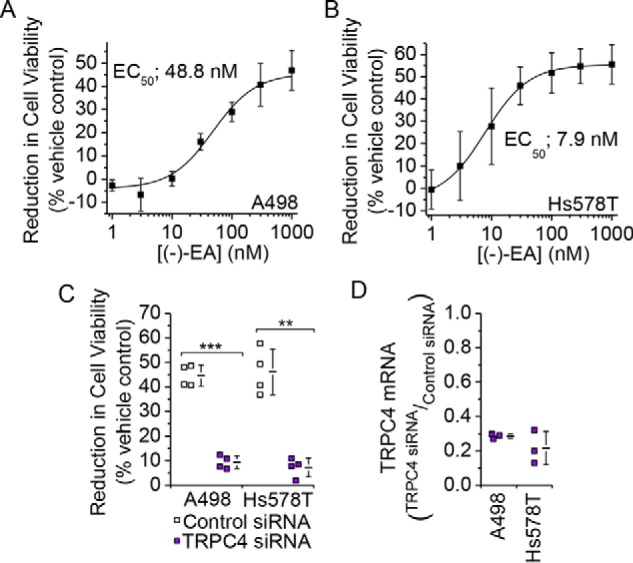
**TRPC4 is important for (−)-EA-evoked cytotoxicity.** *A* and *B*, reductions in A498 and Hs578T cell viabilities caused by 6-h exposures to (−)-EA relative to vehicle control as determined by WST-1 assay. *Error bars* indicate S.D., and the fitted curves are Hill equations (*N* = 3). *C*, (−)-EA-evoked reductions in cell viabilities 72 h after transfection with control siRNA or TRPC4 siRNA (*N* = 4) (**, *p* < 0.01; ***, *p* < 0.001; two-sample *t* test). *D*, knockdown of TRPC4 mRNA. Quantitative RT-PCR was used to determine TRPC4 mRNA levels relative to β-actin mRNA. A498 and Hs578T cells had been transfected 48 h earlier with control siRNA or TRPC4-targeted siRNA (*N* = 3). Each data point in *C* and *D* represents a value from an independent experiment with mean values shown alongside. *Error bars* indicate S.D.

To investigate mechanisms of (−)-EA-induced cytotoxicity, we studied two (−)-EA-sensitive cancer cell lines: A498 renal cell carcinoma cells and Hs578T triple negative breast carcinoma cells (Fig. 1, *A* and *B*). In both cell types, knockdown of TRPC4 suppressed (−)-EA-evoked cytotoxicity (Fig. 1, *C* and *D*).

**FIGURE 2.**
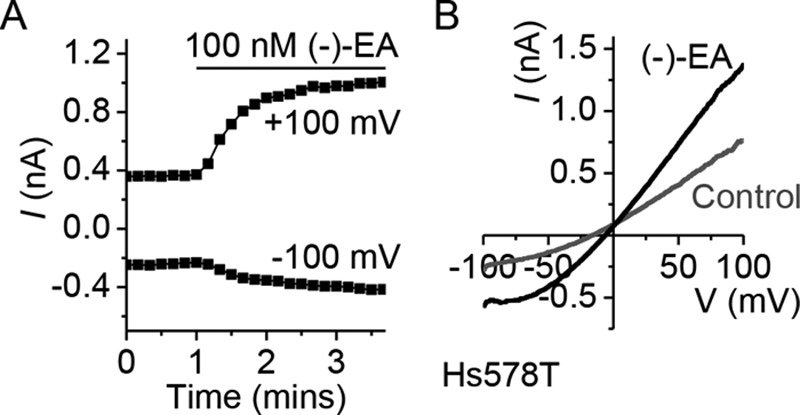
**(−)-EA-activated Hs578T channels are not TRPC4 homomers.** *A*, (−)-EA current in Hs578T. A whole-cell voltage clamp recording of membrane current during ramp changes in membrane voltage from −100 to +100 mV applied every 10 s is shown. Only currents sampled at −100 and +100 mV are displayed. 100 nm (−)-EA was bath-applied as indicated by the *horizontal bar. B*, full current traces during two ramp changes in voltage, one during the initial application of vehicle (DMSO) and the other after application of (−)-EA. Note the absence of a seatlike inflection characteristic of TRPC4 currents (24).

**FIGURE 3.**
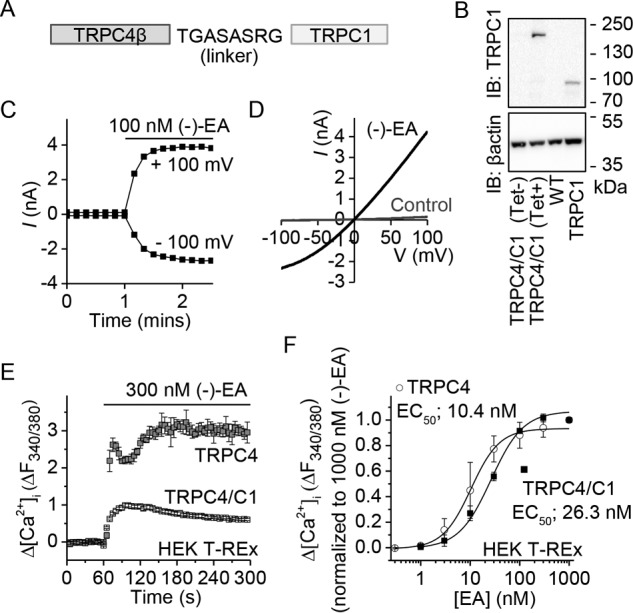
**(−)-EA activates heteromeric channels containing TRPC4 and TRPC1.** *A*, schematic depicting the design of a TRPC4-TRPC1 concatamer with N-terminal TRPC4β (893 amino acids) linked to C-terminal TRPC1 (759 amino acids) via a flexible eight-amino acid linker. *B*, Western blot with anti-TRPC1 antibody confirming tetracycline (*Tet*)-inducible expression of the full-length concatamer protein (predicted size, 191 kDa). Control HEK T-REx cells expressing monomeric TRPC1 protein are provided for comparison. *IB*, immunoblot. *C*, (−)-EA current in HEK T-REx cells expressing the TRPC4-TRPC1 concatamer. A whole-cell voltage clamp recording of membrane current during ramp changes in membrane voltage from −100 to +100 mV applied every 10 s is shown. Only currents sampled at −100 and +100 mV are displayed. 100 nm (−)-EA was bath-applied as indicated by the *horizontal bar. D*, full current traces during two ramp changes in voltage, one during the initial application of vehicle (DMSO) and the other after application of (−)-EA. *E*, example of 96-well plate fura-2 measurements of the change (Δ) in the intracellular Ca^2+^ concentration evoked by (−)-EA in TRPC4- and TRPC4-TRPC1 concatamer (*TRPC4/C1*)-expressing HEK T-REx cells (*n* = 4). *F*, mean data for experiments of the type shown in *E* measured between 1 and 2 min after application of a range of (−)-EA concentrations. *Error bars* indicate S.D., and the fitted curves are Hill equations (*N* = 3).

**FIGURE 4.**
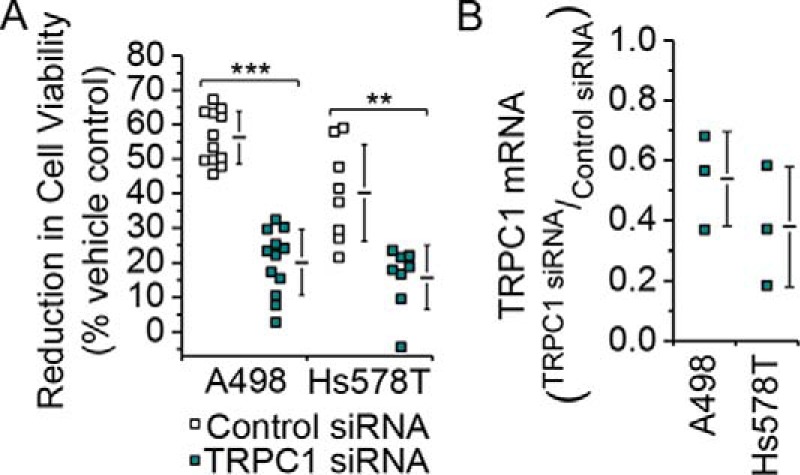
**TRPC1 has a similar role to TRPC4 in (−)-EA-evoked cytotoxicity.** *A*, reductions in A498 and Hs578T cell viabilities caused by 6-h exposures to 300 nm (−)-EA relative to vehicle control as determined by WST-1 assay. Cells had been transfected 72 h earlier with control siRNA or TRPC1 siRNA (A498, *N* = 12; Hs578T, *N* = 8) (**, *p* < 0.01; ***, *p* < 0.001; two-sample *t* test). *B*, knockdown of TRPC1 mRNA. Quantitative RT-PCR was used to determine TRPC1 mRNA levels relative to β-actin mRNA. A498 and Hs578T cells had been transfected 48 h earlier with control siRNA or TRPC1-targeted siRNA (*N* = 3). Each data point in *A* and *B* represents a value from an independent experiment with mean values shown alongside. *Error bars* indicate S.D.

**FIGURE 5.**
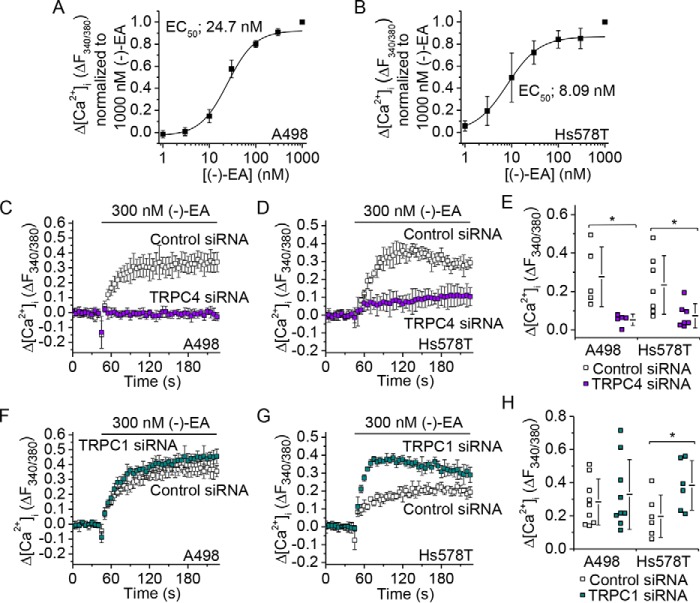
**TRPC1 and TRPC4 make distinct contributions to (−)-EA-evoked Ca^2+^ elevation.** *A* and *B*, mean data for fura-2 measurements of the change (Δ) in the intracellular Ca^2+^ concentration evoked by (−)-EA in A498 and Hs578T cells. Data are plotted as change in the ratio of fluorescence (*F*) emission for 340- and 380-nm excitation wavelengths (Δ*F*_340/380_) normalized to the 1000 nm (−)-EA response. *Error bars* indicate S.D., and the fitted curves are Hill equations (*N* = 3). *C*, *D*, *F*, and *G*, examples of 96-well plate fura-2 measurements of the change in the intracellular Ca^2+^ concentration evoked by 300 nm (−)-EA (*n* = 4). Experiments were paired comparisons of cells transfected with control scrambled siRNA or TRPC4 siRNA (*C* and *D*) or TRPC1 siRNA (*F* and *G*). *E* and *H*, summary for experiments of the type shown in *C*, *D*, *F*, and *G* measured between 1 and 2 min after (−)-EA application. Each data point represents a value from an independent experiment with mean values and *error bars* representing S.D. indicated alongside (in *E*, A498, *N* = 5; Hs578T, *N* = 7; in *H*, A498, *N* = 9; Hs578T, *N* = 6) (*, *p* < 0.05; two-sample *t* test).

Current-voltage relationships of (−)-EA-evoked responses in A498 and Hs578T cells lacked the seatlike inflection of TRPC4 homomers, suggesting that the TRPC4 proteins are in heteromers with TRPC1 (Fig. 2, *A* and *B*) (7). To test the principle of whether (−)-EA can activate TRPC1/TRPC4 heteromers, a stable human embryonic kidney (HEK) 293 cell line inducibly expressing a TRPC4-TRPC1 concatemer was established (Fig. 3, *A* and *B*). Analysis of the current-voltage relationship, which lacked the seatlike inflection of TRPC4 homomers and closely resembled that of A498 and Hs578T cells (Fig. 2*B*) (7), confirmed that the concatemer formed a functional heteromeric channel (Fig. 3, *C* and *D*). Measurement of changes in intracellular Ca^2+^ concentration revealed that although (−)-EA was 2.5-fold less potent at this TRPC4-TRPC1 channel, compared with the TRPC4 homomer, importantly it was able to activate the heteromer at nanomolar concentrations (Fig. 3, *E* and *F*). The involvement of TRPC1 in (−)-EA-induced cytotoxicity was therefore assessed by knockdown of endogenous TRPC1 in the cancer cell lines. Similar to TRPC4 knockdown, TRPC1 knockdown protected against (−)-EA-induced cytotoxicity (Fig. 4, *A* and *B*). The data suggest that (−)-EA evokes cytotoxicity via heteromeric TRPC1/TRPC4 channels and that both TRPC1 and TRPC4 are important.

(−)-EA triggers elevation of intracellular Ca^2+^ (Fig. 5, *A* and *B*), leading to the hypothesis that Ca^2+^ overload mediates the cytotoxicity (7). Consistent with this hypothesis, TRPC4 knockdown dramatically suppressed the elevation in intracellular Ca^2+^ (Fig. 5, *C–E*). In stark contrast, TRPC1 knockdown slightly (A498) or strongly (Hs578T) increased the Ca^2+^ response (Fig. 5, *F–H*). The data suggest that TRPC4 and TRPC1 make opposite contributions to (−)-EA-evoked Ca^2+^ entry with TRPC4 being positive and TRPC1 being null or negative. However, they are both subunits in the same channel, and both are required for (−)-EA-evoked cytotoxicity.

**FIGURE 6.**
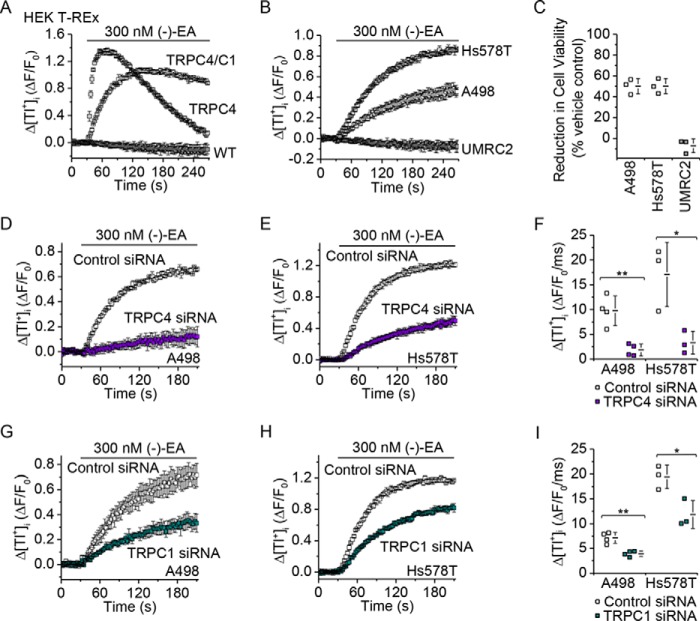
**TRPC1 and TRPC4 make similar contributions to (−)-EA-evoked Tl^+^ entry.** *A*, measurement of Tl^+^ entry through TRPC channels using FluxOR. An example of 96-well plate FluxOR measurements of the change (Δ) in the intracellular Tl^+^ concentration upon application of extracellular Tl^+^ and 300 nm (−)-EA to HEK T-REx cells expressing TRPC4 or TRPC4-TRPC1 concatamer (*TRPC4/C1*) (*n* = 3). The FluxOR measurements are displayed as the fluorescence intensity (*F*) relative to the initial fluorescence intensity (*F*_0_). *B*, FluxOR measurements as for *A* for the cancer cell lines A498, Hs578T, and UMRC2 (*n* = 3). *C*, reductions in cell viabilities caused by 6-h exposures to 300 nm (−)-EA relative to vehicle control as determined by WST-1 assay (*N* = 3). Note the correlation between reduction in cell viability in *C* and the data in *B. D*, *E*, *G*, and *H*, examples of 96-well plate FluxOR measurements upon application of extracellular Tl^+^ and 300 nm (−)-EA in A498 (*D* and *G*) and Hs578T (*E* and *H*) cells (*n* = 3). Experiments were paired comparisons of cells transfected with control scrambled siRNA or TRPC4 siRNA (*D* and *E*) or TRPC1 siRNA (*G* and *H*) (*n* = 3). *F* and *I*, data for experiments of the type shown in *D*, *E*, *G*, and *H* in which the rate of change of *F* was measured between 5 and 35 s after (−)-EA application (A498, *N* = 4; Hs578T, *N* = 3) (*, *p* < 0.05; **, *p* < 0.01; two-sample *t* test). Each data point in *C*, *F*, and *I* represents a value from an independent experiment with mean values indicated alongside. *Error bars* represent S.D.

**FIGURE 7.**
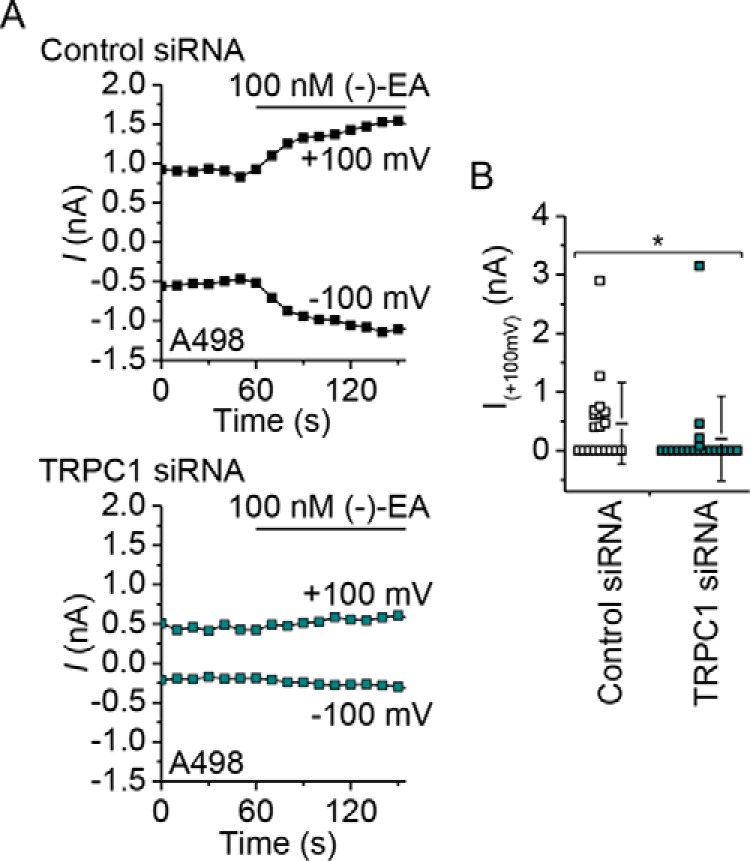
**Suppression of (−)-EA-activated ionic currents by TRPC1 knockdown in A498 cells.** *A*, whole-cell voltage clamp recording of membrane current during ramp changes in membrane voltage from −100 to +100 mV applied every 10 s. Only currents sampled at −100 and +100 mV are displayed. 100 nm (−)-EA was bath-applied as indicated by the *horizontal bar*. A498 cells had been transfected with control siRNA or TRPC1-targeted siRNA 72 h earlier. *B*, current amplitudes at +100 mV with mean values indicated alongside (*N* = 19 cells for each) (*, *p* < 0.05; Mann-Whitney *U* test). *Error bars* represent S.D.

Because Ca^2+^ entry was not correlated with cytotoxicity, we considered an alternative signal mediated by TRPC1/TRPC4 heteromeric channels: monovalent cation entry, which under physiological conditions is Na^+^ entry. To investigate monovalent cation entry in the absence of complicating efflux signals, we measured unidirectional influx of the surrogate cation thallium (Tl^+^), which can be detected by adding Tl^+^ to the extracellular solution and measuring the resulting elevation of intracellular Tl^+^ with the Tl^+^ indicator FluxOR^TM^. This technique reliably detected exogenously expressed TRPC4 homomer and TRPC4-TRPC1 concatemer channel activity (Fig. 6*A*) in HEK 293 cells and (−)-EA responses in A498 and Hs578T cells but not in (−)-EA-insensitive UMRC2 cells (Fig. 6, *B* and *C*). As expected, the A498 and Hs578T responses were suppressed by TRPC4 knockdown (Fig. 6, *D–F*). Moreover, they were suppressed by TRPC1 knockdown (Fig. 6, *G–I*). To further test the role of TRPC1 in monovalent permeability, we performed whole-cell patch clamp recordings where the majority of the ionic current is dependent on flux of monovalent cations. These experiments were challenging because the current responses were commonly not clearly resolved, but again the responses were suppressed by TRPC1 knockdown (Fig. 7). The data suggest that monovalent permeability through TRPC1/TRPC4 heteromers correlates with the cytotoxicity of (−)-EA.

**FIGURE 8.**
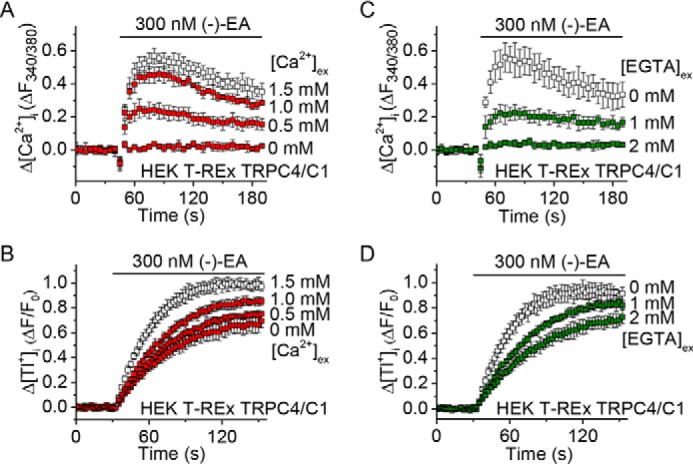
**(−)-EA-evoked Tl^+^ entry in TRPC4/C1-expressing HEK T-REx cells is Ca^2+^-dependent.** *A*, example of 96-well plate fura-2 measurements of the change (Δ) in the intracellular Ca^2+^ concentration evoked by 300 nm (−)-EA in HEK T-REx cells induced to express the TRPC4-TRPC1 concatemer (*TRPC4/C1*). Responses were observed in the presence of 0, 0.5, 1, and 1.5 mm extracellular Ca^2+^ (*[Ca*^*2*+^*]_ex_*) (*n* = 4). *B*, as for *A* but an example of 96-well plate FluxOR measurements of the change (Δ) in the intracellular Tl^+^ concentration upon application of extracellular Tl^+^ and 300 nm (−)-EA (*n* = 4). *C*, as for *A* but in the continuous presence of 1.5 mm extracellular Ca^2+^ plus 0, 1, or 2 mm EGTA to buffer Ca^2+^ (*n* = 4). *D*, as for *B* but in the continuous presence of 1.8 mm extracellular Ca^2+^ plus 0, 1, or 2 mm EGTA to buffer Ca^2+^ (*n* = 4). *Error bars* represent S.D.

**FIGURE 9.**
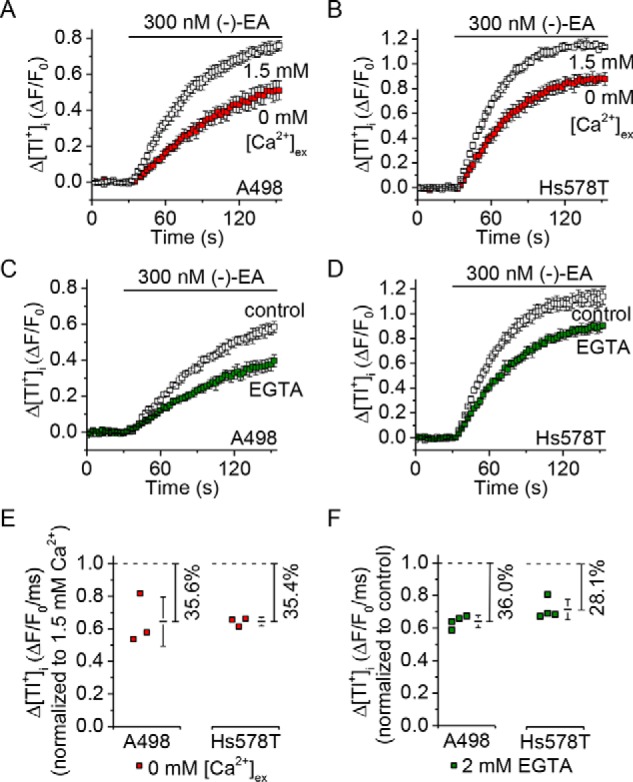
**(−)-EA-evoked Tl^+^ entry in A498 and Hs578T cells is Ca^2+^-dependent.** *A* and *B*, example of 96-well plate FluxOR measurements of the change (Δ) in the intracellular Tl^+^ concentration upon application of extracellular Tl^+^ and 300 nm (−)-EA. Responses were observed in the presence of 0 or 1.5 mm extracellular Ca^2+^ (*[Ca*^*2*+^*]_ex_*) (*n* = 4). *Error bars* indicate S.D. *C* and *D*, as for *A* and *B* but in the continuous presence of 1.8 mm extracellular Ca^2+^ plus 0 or 2 mm EGTA to buffer Ca^2+^ (*n* = 4). *E* and *F*, summary data for experiments of the type shown in *A–D* in which the rate of change of *F* was measured between 5 and 35 s after (−)-EA application. Each data point represents a value from an independent experiment with mean values indicated alongside (in *E*, *N* = 3; in *F*, *N* = 4). Percentages indicate decreases in the mean compared with the respective controls. *Error bars* represent S.D.

The idea that monovalent cation and not Ca^2+^ permeability mediates (−)-EA-evoked cytotoxicity was at odds with our previous observation that decreased extracellular Ca^2+^ concentration afforded protection against (−)-EA-induced cytotoxicity (7). We therefore considered an alternative explanation for this protection in which channel activity (and thus total monovalent cation flux) is Ca^2+^-dependent (9). In support of this explanation, lowering extracellular Ca^2+^ decreased the influx of not only Ca^2+^ but also Tl^+^ through TRPC4-TRPC1 concatemers expressed in HEK 293 cells (Fig. 8) and the endogenous (−)-EA-activated channels in A498 and Hs578T cells (Fig. 9). The data suggest that the Ca^2+^ dependence of channel activity explains the Ca^2+^ dependence of (−)-EA cytotoxicity.

**FIGURE 10.**
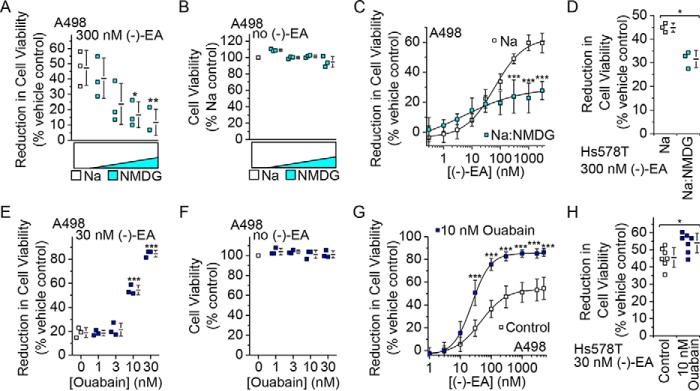
**(−)-EA-evoked cytotoxicity is Na^+^-dependent and potentiated by ouabain.** *A*, reduction of A498 cell viability after 6-h treatment with 300 nm (−)-EA in 150 mm Na^+^ Krebs buffer and increasing replacement of Na^+^ by NMDG^+^ to a maximum of 87.5 mm Na^+^ and 62.5 mm NMDG^+^. Each data point represents a value from an independent experiment with mean values indicated alongside (*N* = 3) (*, *p* < 0.05; **, *p* < 0.01; one-way ANOVA with Bonferroni post hoc test). *Error bars* represent S.D. *B*, as for *A* but in the absence of (−)-EA and shown as cell viability relative to 150 mm Na^+^ Krebs buffer (control) (*N* = 3). *C*, concentration-dependent reduction in A498 cell viability in response to (−)-EA in Krebs buffer containing 150 mm Na^+^ (*Na*) or 100 mm Na^+^ and 50 mm NMDG^+^ (*Na:NMDG*) (*N* = 5). *Error bars* indicate S.D., and the fitted curves are Hill equations with midpoints of 63.5 (Na^+^) and 4.5 nm (Na^+^ and NMDG^+^) (***, *p* < 0.001; one-way ANOVA with post hoc Bonferroni test). *D*, reduction of Hs578T cell viability after 6-h treatment with 300 nm (−)-EA in 150 mm Na^+^ (*Na*) or 100 mm Na^+^ and 50 mm NMDG^+^ (*Na:NMDG*) (*N* = 3) (*, *p* < 0.05; two-sample *t* test). *Error bars* represent S.D. *E*, reduction of A498 cell viability after 6-h treatment with 30 nm (−)-EA plus 0, 1, 3, 10, and 30 nm ouabain 30 min prior to (−)-EA and during (−)-EA exposure (*N* = 3) (***, *p* < 0.001; one-way ANOVA with post hoc Bonferroni test). *Error bars* represent S.D. *F*, as for *E* but in the absence of (−)-EA and shown as cell viability relative to 0 nm ouabain (control) (*N* = 3). *G*, reduction of A498 cell viability after 6-h treatment with (−)-EA in vehicle (control) and in the presence of 10 nm ouabain (*N* = 3). *Error bars* indicate S.D., and the fitted curves are Hill equations with midpoints of 45.2 (control) and 23.5 nm (ouabain) (***, *p* < 0.001; one-way ANOVA with Bonferroni post hoc test). *H*, reduction of Hs578T cell viability after 6-h treatment with 30 nm (−)-EA in vehicle (control) and the presence of 10 nm ouabain (*N* = 7) (*, *p* < 0.05; two-sample *t* test). *Error bars* represent S.D.

To investigate whether Na^+^ is the trigger for (−)-EA-induced cytotoxicity, Na^+^ was substituted progressively by the larger non-permeant cation *N*-methyl-d-glucamine (NMDG^+^). Strikingly, this resulted in progressive protection against cytotoxicity (Fig. 10*A*) evident at concentrations at which the switch to NMDG^+^ had no effect in the absence of (−)-EA (Fig. 10*B*). Protection did not result from a decrease in the potency of (−)-EA because partial substitution of Na^+^ by NMDG^+^ improved the EC_50_ for (−)-EA-induced cytotoxicity from 63.5 to 29.6 nm (Fig. 10*C*). Similar protective effects against (−)-EA cytotoxicity were observed for Hs578T cells (Fig. 10*D*). The data suggest that Na^+^ mediates (−)-EA-evoked cytotoxicity.

A feature of the (−)-EA effect on cell viability was that only about half of all cells died during the 6-h exposure to (−)-EA (Fig. 1, *A* and *B*). Although this is a relatively short time, we considered whether the cells contained a mechanism for protecting themselves against excess sustained Na^+^ entry and its cytotoxicity. Speculating that Na^+^/K^+^-ATPase might provide protection by counteracting Na^+^ influx by ATP-dependent Na^+^ extrusion, we inhibited Na^+^/K^+^-ATPase with ouabain and tested a concentration of (−)-EA close to the EC_50_ for A498 cytotoxicity (Fig. 1*A*). Ouabain conferred a marked increase in cytotoxicity such that at 30 nm almost 85% of the cells now died within the 6 h (Fig. 10*E*). Ouabain alone was without effect at ≤30 nm (Fig. 10*F*). Ouabain not only increased the number of dead cells but also the potency of (−)-EA; 10 nm ouabain shifted the EC_50_ from 45.2 to 23.5 nm (Fig. 10*G*). A similar but smaller effect of ouabain was observed on (−)-EA-induced Hs578T cell death (Fig. 10*H*). The data suggest that Na^+^/K^+^-ATPase protects against (−)-EA-evoked cytotoxicity.

## Discussion

In this study, we show the surprising finding that (−)-EA cytotoxicity results from Na^+^, not Ca^2+^, influx through TRPC4-containing channels. This led us to reveal that Na^+^/K^+^-ATPase protects against the cytotoxicity and that ouabain, a Na^+^/K^+^-ATPase inhibitor, can enhance it. Reduced Na^+^/K^+^-ATPase activity has been observed in human renal cell carcinomas associated with reduced β_1_-subunit (10, 11). Therefore, if (−)-EA is pursued as a therapeutic direction, it might be worthwhile to monitor Na^+^/K^+^-ATPase as an indicator of potential (−)-EA sensitivity. Additionally co-administration of a Na^+^/K^+^-ATPase inhibitor, such as digoxin, could be an approach to provoke or increase (−)-EA sensitivity and perhaps augment its anticancer effects.

A key feature of (−)-EA cytotoxicity is its rapidity (7). Therefore, fast signaling mechanisms are presumably pivotal, and slower mechanisms such as transcription, translation, and protein degradation are less relevant. Accordingly, we focused on effects of (−)-EA within 6 h. Based on prior work and data shown here, we suggest the following sequence of events. (−)-EA binds to TRPC1/TRPC4 ion channels in the plasma membrane, which increases the probability of channel opening and leads to sustained increases in Ca^2+^ and Na^+^ influx through the channel pore. TRPC1 suppresses Ca^2+^ permeability (12); therefore, the global cytosolic Ca^2+^ elevation is relatively small and non-toxic. Nevertheless, Ca^2+^ concentration elevations near the channels facilitate their activity (Fig. 9). The Na^+^ influx elevates the global cytosolic Na^+^ concentration, stimulating Na^+^/K^+^-ATPase, a Na^+^-dependent pump that utilizes ATP to drive Na^+^ extrusion. In some cells, activity of the pump is insufficient to prevent Na^+^ accumulating to a cytotoxic threshold, so these cells die. In other cells, pump activity is sufficient, and the cells successfully resist (−)-EA. Blockade of the pump negates protection afforded by the pump, so more cells die. We suggest therefore that (−)-EA-dependent cytotoxicity depends on (i) sufficient functional TRPC1/TRPC4 heteromeric channels at the plasma membrane and (ii) insufficient Na^+^/K^+^-ATPase activity to counteract the sustained cytosolic Na^+^ elevation caused by frequent TRPC1/TRPC4 channel opening.

**FIGURE 11.**
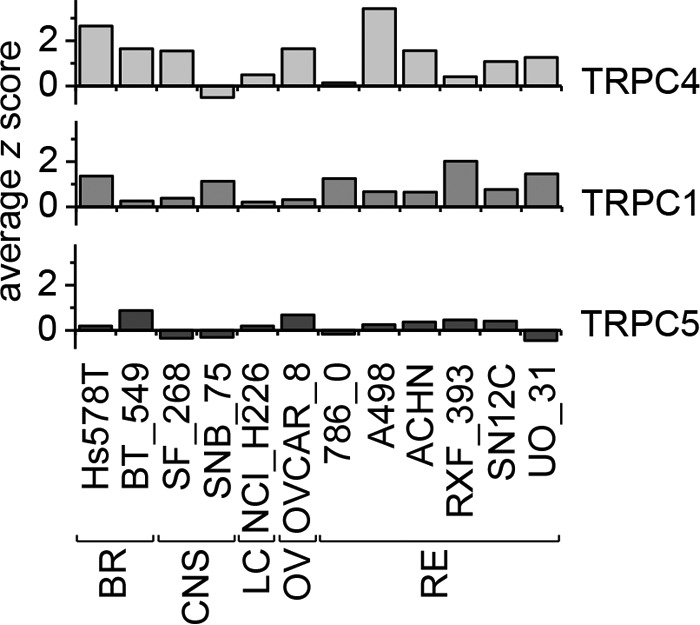
**Comparison of TRPC4, TRPC1, and TRPC5 gene transcript levels in (−)-EA-sensitive NCI60 cell lines.** TRPC4, TRPC1, and TRPC5 gene transcript data from the CellMiner database (13, 14) for NCI60 cell lines identified by Ratnayake *et al.* (1) to be killed by (−)-EA are shown. The z scores reflect the number of standard deviations from the mean. *BR*, breast; *CNS*, central nervous system; *LC*, non-small cell lung; *OV*, ovarian; *RE*, renal.

Previous work has focused on TRPC4 as the target for (−)-EA, and our data support this mechanism (Fig. 1*C*). Although a contribution of TRPC1 to the (−)-EA-evoked ionic current was suggested previously (7), the data shown here provide the first evidence that TRPC1 is also important for (−)-EA-evoked cytotoxicity. Analysis of CellMiner^TM^ gene transcript data for NCI60 cell lines (13, 14) reveals positive z scores indicative of elevated TRPC1 expression in all 12 (−)-EA-sensitive cell lines studied by Ratnayake *et al.* (1) (Fig 11). Therefore, involvement of TRPC1 in (−)-EA cytotoxicity is unlikely to be restricted to the two cell lines tested in our study. It is unclear whether a particular property of TRPC1 is important for (−)-EA cytotoxicity. TRPC1 slightly reduces (−)-EA potency, so it does not contribute substantially by altering the binding or efficacy of (−)-EA (Fig. 3*F*). It is possible that it contributes simply by increasing the total number of TRPC proteins available for channel formation. However, this would only be advantageous if the Na^+^ conductance and opening probability of heteromeric channels were equal to or greater than those of homomeric TRPC4 channels. This comparison has yet to be made for TRPC4 channels, but TRPC1 markedly reduces the unitary conductance of the closely related TRPC5 channels (15–17). Also, although the influence of TRPC4 on the trafficking of TRPC1 is well documented (18, 19), details of the reverse relationship and therefore the influence of TRPC1 on cell surface channel density are lacking.

The mechanism by which Na^+^ mediates (−)-EA cytotoxicity is currently unknown. Our findings nevertheless suggest a hypothesis whereby excess sustained Na^+^ entry causes ATP depletion by forcing Na^+^/K^+^-ATPase into overdrive. Na^+^/K^+^-ATPases are recognized as the major energy consumer in the brain (20), and this may also be true in cancer cells because Na^+^/K^+^-ATPases are the workhorses underlying Ca^2+^, H^+^, K^+^, Na^+^, Cl^−^, water, and volume homeostasis. Inhibitors of Na^+^/K^+^-ATPase would suppress this ATP utilization but at the cost of failed ionic, osmotic, and volume homeostasis.

In summary, this study has revealed a surprising biophysical phenomenon whereby Ca^2+^, the supreme signaling ion of eukaryotic biology, is not the mediator of (−)-EA-evoked cytotoxicity even though the target channels are Ca^2+^-permeable and the intracellular Ca^2+^ concentration does rise in response to (−)-EA. Instead it is the Na^+^ permeability of the channel that mediates the cytotoxicity. How Na^+^ causes such an effect remains an open question, but what has become apparent is that Na^+^/K^+^-ATPase protects cancer cells against the Na^+^ influx evoked by (−)-EA. Na^+^/K^+^-ATPase depletion, which occurs in cancer, could be a cofactor in (−)-EA sensitivity, and pharmacological inhibition of the ATPase strikingly increased the vulnerability of cancer cells to (−)-EA. Recently, Na^+^/K^+^ ATPase inhibitors, which have long been used in cardiovascular therapy, have been suggested to have anticancer effects (21). Although such inhibitors cause adverse effects, they are commonly judged to be tolerable. Because (−)-EA also has adverse effects (6), efforts are needed to determine whether they would preclude translation toward a new anticancer therapy.

## Experimental Procedures

### 

#### 

##### Materials

(−)-EA was purchased from PhytoLab, and a 10 mm stock solution was prepared in DMSO (stored at −80 °C). To minimize (−)-EA aggregation, 0.01% Pluronic acid was included in experimental solutions as a dispersing agent. Ouabain octahydrate, dissolved in medium at 0.5 mm, was purchased from Sigma-Aldrich.

##### Intracellular Calcium (Ca^2+^) (Fura-2) or Thallium (Tl^+^) (FluxOR) Measurements

For intracellular Ca^2+^ or Tl^+^ assays, cells were plated at 80–90% confluence in 96-well plates 24 h prior to recordings (4 × 10^4^ HEK T-REx, 1.8 × 10^4^ A498, 2.2 × 10^4^ Hs578T, and 1.2 × 10^4^ UMRC2 cells). To measure intracellular Ca^2+^, cells were incubated for 1 h at 37 °C in standard bath solution (SBS; 135 mm NaCl, 5 mm KCl, 1.2 mm MgCl_2_, 1.5 mm CaCl_2_, 8 mm glucose, and 10 mm HEPES (pH titrated to 7.4 using NaOH)) containing 2 μm fura-2 acetoxymethyl ester (Molecular Probes) with 0.01% Pluronic acid. Cells were washed with SBS and incubated at room temperature for 20 min prior to recordings. To measure Tl^+^ influx, cells were loaded with the FluxOR dye for 1 h at room temperature, transferred to assay buffer, and stimulated with a Tl^+^-containing K^+^-free solution according to the manufacturer's instructions (Molecular Probes). To assess the impact of extracellular Ca^2+^ on channel function using the FluxOR assay, calcium-free assay buffer (136.9 mm NaCl, 0.33 mm Na_2_HPO_4_, 4.17 mm NaHCO_3_, 5.33 mm KCl, 0.44 mm KH_2_PO_4_, 0.49 mm MgCl_2_, 0.41 mm MgSO_4_, 5.55 mm d-glucose, and 20 mm HEPES (pH titrated to 7.4 using NaOH)) and Ca^2+^-free stimulus buffer (140 mm sodium gluconate, 2.5 mm potassium gluconate, 1 mm magnesium gluconate, and 20 mm HEPES (pH titrated to 7.4 with NaOH)) to which a range of CaCl_2_ and calcium gluconate concentrations were supplemented, respectively, were used. Measurements were made on a fluorescence plate reader (Flexstation, Molecular Devices). Fura-2 was excited at 340 and 380 nm, and emitted light was collected at 510 nm with measurements shown as the change in fluorescence (*F*) ratio (Δ*F*_340/380_). FluxOR was excited at 485 nm, emitted light was collected at 520 nm, and measurements were expressed as a ratio increase over baseline (*F*/*F*_0_) with vehicle (DMSO) values subtracted from (−)-EA values at each time point (Δ*F*/*F*_0_).

##### Determination of Cell Viability

For cell viability assays, cells were seeded onto 96-well plates 24 h prior to WST-1 measurements (4 × 10^3^ A498, 7.5 × 10^3^ Hs578T, 5 × 10^3^ UMRC2, and 1.5 × 10^4^ HEK T-REx cells). The cell proliferation reagent WST-1 (Roche Applied Science) was used according to the manufacturer's instructions. Reduction of the tetrazolium salt WST-1 to formazan by mitochondrial dehydrogenases was determined by absorbance measurement at 450 nm. Cell death, therefore loss of mitochondrial dehydrogenase activity, was inferred from a decrease in this reaction. Background absorbance at the reference wavelength 655 nm was subtracted (Bio-Rad iMark^TM^ microplate absorbance reader). Krebs buffer (125 mm NaCl, 3.8 mm KCl, 1.5 mm MgSO_4_, 1.2 mm KH_2_PO_4_, 25 mm NaHCO_3_, 1.2 mm CaCl_2_, and 8 mm glucose (pH 7.4)) instead of medium was used to facilitate substitution of NaCl for NMDG.

##### Cell Culture and Expression Systems

HEK T-REx^TM^-293 (HEK T-REx, Invitrogen) cell lines stably expressing human TRPC4β or human TRPC3 under a tetracycline-inducible promoter have been described previously (7, 22). An HEK T-REx cell line stably expressing a human TRPC4β-TRPC1 chimera was generated similarly and validated by Western blotting and biophysical analysis. 1 μg/ml tetracycline was added to cells for 24 h to induce channel expression. These HEK 293 cells were cultured in Dulbecco's modified Eagle's medium GlutaMAX with 4.5 g/liter d-glucose and pyruvate (Gibco) supplemented with the selection antibiotics 5 μg/ml blasticidin and 400 μg/ml Zeocin (InvivoGen). A498 cells were from ATCC and were cultured in minimum essential Eagle's medium with Earle's balanced salt solution, l-glutamine, and 2.2 g/liter NaHCO_3_ (PAN-biotech, Germany) supplemented with sodium pyruvate. Hs875T cells (ATCC) and UMRC2 cells (a generous gift from Prof. Eamonn Maher, University of Cambridge) were cultured in RPMI 1640 medium with l-glutamine (Gibco). All media were supplemented with 10% FBS, 50 units/ml penicillin, and 0.5 mg/ml streptomycin and grown at 37 °C in a 5% CO_2_ incubator.

Cells were transfected at 90% confluence with 40 nm siRNA using Lipofectamine 2000 (Invitrogen) in Opti-MEM (Gibco) according to the manufacturer's instructions. Medium was replaced after 5 h, and cells were used for experimentation 48–72 h after transfection. siRNAs used were as follows: Silencer® Negative Control Number 1 (catalog number AM4611), human TRPC1 (identification number 138932) from Ambion, and ON-TARGETplus Non-targeting Pool (catalog number D-001810-10-05) and human TRPC4 (catalog number L-0065-10-01) from Dharmacon.

##### Concatemer Construct

Human TRPC4 and TRPC1 were cloned upstream and downstream, respectively, of a four-amino acid linker (ASAS) flanked by AgeI and SacII restriction endonuclease sites that had previously been introduced into pcDNA^TM^4/TO (22). TRPC4β, including an N-terminal Kozak sequence, was inserted between BamHI and AgeI restriction sites using hTRPC4β/pcDNA4/TO (7) as a PCR template (forward primer, 5′-AGTCGGATCCGCCACCATGGCTCAGTTCTATTACAAAAG-3′; reverse primer, 5′-AGTTACCGGTCAATCTTGTGGTCACGTAATCTTC-3′). TRPC1 was inserted between SacII and XbaI restriction sites using hTRPC1/pIRES (23) as a PCR template (forward primer, 5′-ACTCCGCGGCATGATGGCGGCCCTG-3′; reverse primer, 5′-AGTCTCTAGATTAATTTCTTGGATAAAACATAGCATATTTAG-3′).

##### Electrophysiology

Borosilicate glass capillaries with an outside diameter of 1 mm and an inside diameter of 0.58 mm (Harvard Apparatus) were used as the basis for patch pipettes. Pipettes were pulled using a PP-830 vertical two-stage pipette puller (Narishige, Tokyo, Japan). Pipette resistances after fire polishing and filling with pipette solution were 2–4 megaohms. Pipettes were mounted on a CV-4 head stage (Molecular Devices) connected to a three-way coarse manipulator and micromanipulator (Mitutoyo, Japan). The electrode was an Ag/AgCl wire. Electrical signals were amplified and recorded using an Axopatch 200B amplifier and pCLAMP 10 software (Molecular Devices). Data were filtered at 1 kHz and sampled digitally at 3 kHz via a Digidata 1440A analogue-to-digital converter (Molecular Devices). Analysis was performed offline using Clampfit 10.2 (Molecular Devices) and Origin 8.6 software (OriginLab). Recordings were made at room temperature and with SBS as the extracellular solution. The standard patch pipette solution contained 145 mm CsCl, 2 mm MgCl_2_, 10 mm HEPES, 1 mm EGTA (free acid), 5 mm ATP (sodium salt), and 0.1 mm Na.GTP (sodium salt) (titrated to pH 7.2 with CsOH).

##### Quantitative Real Time Reverse Transcription (RT)-PCR

Total RNA was extracted using TRI Reagent (Sigma-Aldrich) followed by DNase I (Ambion) treatment. 1 μg of total RNA was used for RT using oligo(dT) primers (Ambion) and avian myeloblastosis virus reverse transcriptase enzyme (Ambion). Real time PCR was conducted using a Roche Applied Science LightCycler. PCR primers used were as follows: β-actin: forward, 5′-TCGAGCAAGAGATGGC-3′; reverse, 5′-TGAAGGTAGTTTCGTGGATG-3′; human TRPC1: forward, 5′-TTAGCGCATGTGGCAA-3′; reverse, 5′-CCACTTACTGAGGCTACTAAT-3′; and human TRPC4: forward, 5′-ATTAGCTTCACGGGGT-3′; reverse, 5′-GTTTAGATCATAGTCTATACTAGAGTCC-3′.

##### Western Blotting for TRP Proteins

Cells were harvested in lysis buffer (10 mm Tris (pH 7.4), 150 mm NaCl, 0.5 mm EDTA, and 0.5% Nonidet P40 substitute) containing protease inhibitor cocktail (Sigma). Equal protein amounts were loaded on 8% gels and resolved by electrophoresis. Samples were transferred to PVDF membranes and labeled overnight with anti-TRPC1 (1:1000; NeuroMab clone 1F1) or anti-β-actin (200 ng/ml; Santa Cruz Biotechnology). Horseradish peroxidase-donkey anti-mouse secondary antibody (Jackson ImmunoResearch Laboratories) and SuperSignal Pico/Femto detection reagents (Pierce) were used for visualization.

##### Statistical Analysis

Averaged data are shown as mean ± S.D. Data were produced in pairs and analyzed statistically with two-sample *t* tests or one-way ANOVA using OriginPro 8.6 software (OriginLab). Electrophysiological data (Fig. 7) were analyzed with the Mann-Whitney *U* test. Statistically significant differences are indicated by * (*p* < 0.05), ** (*p* < 0.01), or *** (*p* < 0.001). “*N*” is used to denote the number of independent data points, each point representing the mean value from a single independent experiment. Representative traces contain “*n*” replicates from within a single experiment.

## Author Contributions

M. J. L. conducted most of the experiments, analyzed the results, and wrote the paper. H. J. G. contributed to the data presented in Figs. 4 and 5. K. M. conducted the experiments in Figs. 2 and 7. K. E. M. and N. M. B. established the HEK T-REx TRPC4/C1 cell line (Fig. 3). H. N. R. performed patch clamp characterization of the heteromeric channel (Fig. 3, *C* and *D*). N. S. V. made an intellectual contribution to renal cancer and cancer cells. D. J. B. conceived the idea and raised funds for the project and wrote the paper with M. J. L.

## Footnotes

2 The abbreviations used are:

(−)-EA: (−)-englerin A
TRPC: transient receptor potential canonical
NMDG: *N*-methyl-d-glucamine
EC_50_: half-maximal effective concentration
SBS: standard bath solution
ANOVA: analysis of variance.

## References

[1] Ratnayake R., Covell D., Ransom T. T., Gustafson K. R., and Beutler J. A. (2009) Englerin A, a selective inhibitor of renal cancer cell growth, from *Phyllanthus engleri*. Org. Lett. 11, 57–60 19061394 10.1021/ol802339w PMC2651161

[2] Fash D. M., Peer C. J., Li Z., Talisman I. J., Hayavi S., Sulzmaier F. J., Ramos J. W., Sourbier C., Neckers L., Figg W. D., Beutler J. A., and Chain W. J. (2016) Synthesis of a stable and orally bioavailable englerin analogue. Bioorg. Med. Chem. Lett. 26, 2641–2644 27107948 10.1016/j.bmcl.2016.04.016 PMC4862412

[3] Li Z., Nakashige M., and Chain W. J. (2011) A brief synthesis of (−)-englerin A. J. Am. Chem. Soc. 133, 6553–6556 21476574 10.1021/ja201921j

[4] López-Suárez L., Riesgo L., Bravo F., Ransom T. T., Beutler J. A., and Echavarren A. M. (2016) Synthesis and biological evaluation of new (−)-englerin analogues. ChemMedChem 11, 1003–1007 27005578 10.1002/cmdc.201600040 PMC4926265

[5] Radtke L., Willot M., Sun H., Ziegler S., Sauerland S., Strohmann C., Fröhlich R., Habenberger P., Waldmann H., and Christmann M. (2011) Total synthesis and biological evaluation of (−)-englerin A and B: synthesis of analogues with improved activity profile. Angew. Chem. Int. Ed. Engl. 50, 3998–4002 21472928 10.1002/anie.201007790

[6] Carson C., Raman P., Tullai J., Xu L., Henault M., Thomas E., Yeola S., Lao J., McPate M., Verkuyl J. M., Marsh G., Sarber J., Amaral A., Bailey S., Lubicka D., et al (2015) Englerin A agonizes the TRPC4/C5 cation channels to inhibit tumor cell line proliferation. PLoS One 10, e0127498 26098886 10.1371/journal.pone.0127498 PMC4476799

[7] Akbulut Y., Gaunt H. J., Muraki K., Ludlow M. J., Amer M. S., Bruns A., Vasudev N. S., Radtke L., Willot M., Hahn S., Seitz T., Ziegler S., Christmann M., Beech D. J., and Waldmann H. (2015) (−)-Englerin A is a potent and selective activator of TRPC4 and TRPC5 calcium channels. Angew. Chem. Int. Ed. Engl. 54, 3787–3791 25707820 10.1002/anie.201411511 PMC7116557

[8] Sourbier C., Scroggins B. T., Ratnayake R., Prince T. L., Lee S., Lee M. J., Nagy P. L., Lee Y. H., Trepel J. B., Beutler J. A., Linehan W. M., and Neckers L. (2013) Englerin A stimulates PKCθ to inhibit insulin signaling and to simultaneously activate HSF1: pharmacologically induced synthetic lethality. Cancer Cell 23, 228–237 23352416 10.1016/j.ccr.2012.12.007 PMC3574184

[9] Plant T. D., and Schaefer M. (2003) TRPC4 and TRPC5: receptor-operated Ca^2+^-permeable nonselective cation channels. Cell Calcium 33, 441–450 12765689 10.1016/s0143-4160(03)00055-1

[10] Rajasekaran S. A., Ball W. J. Jr, Bander N. H., Liu H., Pardee J. D., and Rajasekaran A. K. (1999) Reduced expression of β-subunit of Na,K-ATPase in human clear-cell renal cell carcinoma. J. Urol. 162, 574–580 10411090

[11] Selvakumar P., Owens T. A., David J. M., Petrelli N. J., Christensen B. C., Lakshmikuttyamma A., and Rajasekaran A. K. (2014) Epigenetic silencing of Na,K-ATPase β1 subunit gene ATP1B1 by methylation in clear cell renal cell carcinoma. Epigenetics 9, 579–586 24452105 10.4161/epi.27795 PMC4121368

[12] Storch U., Forst A. L., Philipp M., Gudermann T., and Mederos y Schnitzler M. (2012) Transient receptor potential channel 1 (TRPC1) reduces calcium permeability in heteromeric channel complexes. J. Biol. Chem. 287, 3530–3540 22157757 10.1074/jbc.M111.283218 PMC3271006

[13] Reinhold W. C., Sunshine M., Liu H., Varma S., Kohn K. W., Morris J., Doroshow J., and Pommier Y. (2012) CellMiner: a web-based suite of genomic and pharmacologic tools to explore transcript and drug patterns in the NCI-60 cell line set. Cancer Res. 72, 3499–3511 22802077 10.1158/0008-5472.CAN-12-1370 PMC3399763

[14] Shankavaram U. T., Varma S., Kane D., Sunshine M., Chary K. K., Reinhold W. C., Pommier Y., and Weinstein J. N. (2009) CellMiner: a relational database and query tool for the NCI-60 cancer cell lines. BMC Genomics 10, 277 19549304 10.1186/1471-2164-10-277 PMC2709662

[15] Alfonso S., Benito O., Alicia S., Angélica Z., Patricia G., Diana K., and Vaca L. (2008) Regulation of the cellular localization and function of human transient receptor potential channel 1 by other members of the TRPC family. Cell Calcium 43, 375–387 17850866 10.1016/j.ceca.2007.07.004

[16] Shi J., Ju M., Abramowitz J., Large W. A., Birnbaumer L., and Albert A. P. (2012) TRPC1 proteins confer PKC and phosphoinositol activation on native heteromeric TRPC1/C5 channels in vascular smooth muscle: comparative study of wild-type and TRPC1−/− mice. FASEB J. 26, 409–419 21968068 10.1096/fj.11-185611 PMC3250247

[17] Strübing C., Krapivinsky G., Krapivinsky L., and Clapham D. E. (2001) TRPC1 and TRPC5 form a novel cation channel in mammalian brain. Neuron 29, 645–655 11301024 10.1016/s0896-6273(01)00240-9

[18] Dietrich A., Fahlbusch M., and Gudermann T. (2014) Classical transient receptor potential 1 (TRPC1): channel or channel regulator? Cells 3, 939–962 25268281 10.3390/cells3040939 PMC4276908

[19] Kim J., Kwak M., Jeon J. P., Myeong J., Wie J., Hong C., Kim S. Y., Jeon J. H., Kim H. J., and So I. (2014) Isoform- and receptor-specific channel property of canonical transient receptor potential (TRPC)1/4 channels. Pflugers Arch. 466, 491–504 23948741 10.1007/s00424-013-1332-y

[20] Engl E., and Attwell D. (2015) Non-signalling energy use in the brain. J. Physiol. 593, 3417–3429 25639777 10.1113/jphysiol.2014.282517 PMC4560575

[21] Alevizopoulos K., Calogeropoulou T., Lang F., and Stournaras C. (2014) Na^+^/K^+^ ATPase inhibitors in cancer. Curr. Drug Targets 15, 988–1000 25198786

[22] Naylor J., Minard A., Gaunt H. J., Amer M. S., Wilson L. A., Migliore M., Cheung S. Y., Rubaiy H. N., Blythe N. M., Musialowski K. E., Ludlow M. J., Evans W. D., Green B. L., Yang H., You Y., et al (2016) Natural and synthetic flavonoid modulation of TRPC5 channels. Br. J. Pharmacol. 173, 562–574 26565375 10.1111/bph.13387 PMC4728423

[23] Xu S. Z., Muraki K., Zeng F., Li J., Sukumar P., Shah S., Dedman A. M., Flemming P. K., McHugh D., Naylor J., Cheong A., Bateson A. N., Munsch C. M., Porter K. E., and Beech D. J. (2006) A sphingosine-1-phosphate-activated calcium channel controlling vascular smooth muscle cell motility. Circ. Res. 98, 1381–1389 16675717 10.1161/01.RES.0000225284.36490.a2 PMC2648505

[24] Schaefer M., Plant T. D., Obukhov A. G., Hofmann T., Gudermann T., and Schultz G. (2000) Receptor-mediated regulation of the nonselective cation channels TRPC4 and TRPC5. J. Biol. Chem. 275, 17517–17526 10837492 10.1074/jbc.275.23.17517

